# Adaptive and Efficient Mixture-Based Representation for Range Data

**DOI:** 10.3390/s20113272

**Published:** 2020-06-08

**Authors:** Minghe Cao, Jianzhong Wang, Li Ming

**Affiliations:** 1 School of Mechatronical Engineering, Beijing Institute of Technology, Beijing 100081, China; 3120160104@bit.edu.cn; 2 School of Automation, Beijing Institute of Technology, Beijing 100081, China; mingllove@bit.edu.cn

**Keywords:** gaussian mixture model, environment representation, hierarchical structure, point cloud data

## Abstract

Modern range sensors generate millions of data points per second, making it difficult to utilize all incoming data effectively in real time for devices with limited computational resources. The Gaussian mixture model (GMM) is a convenient and essential tool commonly used in many research domains. In this paper, an environment representation approach based on the hierarchical GMM structure is proposed, which can be utilized to model environments with weighted Gaussians. The hierarchical structure accelerates training by recursively segmenting local environments into smaller clusters. By adopting the information-theoretic distance and shape of probabilistic distributions, weighted Gaussians can be dynamically allocated to local environments in an arbitrary scale, leading to a full adaptivity in the number of Gaussians. Evaluations are carried out in terms of time efficiency, reconstruction, and fidelity using datasets collected from different sensors. The results demonstrate that the proposed approach is superior with respect to time efficiency while maintaining the high fidelity as compared to other state-of-the-art approaches.

## 1. Introduction

Range data have widely been used in applications including medical imaging, object modeling, and robotics state estimation. Modern range sensors (e.g., LiDAR, RGB-D cameras) generate millions of data points per second, making it difficult to utilize all incoming data effectively in real time for devices with limited computational resources. Along with the impact of sensor noises, it is rarely feasible to operate directly on the raw point measurements obtained from the sensors in robotics applications.

Point cloud representation techniques are developed to address this problem. The direct dense representation involves stitching point clouds from different poses of frames, and downsampling data via various filters. Other techniques include discretizing the environment into grids or voxels. The Occupancy Grid Map was first proposed by Elfes [1] for the perception and navigation of mobile robots. The environment was represented as discretized binary grids whose status are either occupied or not occupied. Ryde et al. [2] extended the Occupancy Grid Map to three dimensions by aligning range data stored in occupied voxel lists. Li [3] applied the Occupancy Grid Map to the urban scenario. OctoMap [4] is one of the most popular volumetric environment models because it can be flexibly deployed in multiresolution. While downsampling and simple discretization may result in loss of information, the Normal Distributions Transform (NDT) [5] assigns a normal distribution to each grid cell to model the distribution of local environments, which was extended to 3D environments by Magnusson [6]. The NDT approach and its variants provide a higher fidelity of representation compared to occupancy methods. However, the discretized grids and voxels are independent and set at a fixed resolution, which may lead to inconsistency at the boundaries. Furthermore, it is hard to deploy these models in large scale environments due to the memory requirements. Other than traditional methods, approaches based on deep learning [7,8,9] have also been proved efficient and robust in facilitating representation.

As a result of the flexibility to model distributions whose parametric forms are unknown, the Gaussian Mixture Model (GMM) is a convenient and essential probability model used in many research domains, from image processing to machine learning [10,11,12,13,14]. Approaches have been proposed to address the various problems associated with GMMs, such as problems involving environment representation [15,16], registration [17,18,19,20,21,22], mapping [23,24,25], localization [26], and planning [25]. Jian et al. [18] and Segal et al. [19] converted a point cloud into a GMM by assigning a covariance matrix to every point for registration. The difference is that the covariance matrices in [18] are isotropic, whereas Segal [19] considered that each point distributes with a high covariance along its local plane and a low covariance in the normal surface direction. Though these construction methods minimized the cost in the setup step, the computational cost in the registration step increased since the number of components is much greater than in the training method. Other approaches, such as those described in [15,16], model environments by training point cloud data into anisotropic Gaussian components using the Expectation–Maximization (EM) algorithm.

Even though the anisotropic GMM representation approach has been proved to be memory efficient, it still has several drawbacks. Firstly, as a result of the fact that there is no closed form solution to analytically solve GMM parameters when fitting a point cloud, approaches like EM are commonly used to give an estimation. However, the time complexity of the EM algorithm is O(N×C) where *N* is the size of the environment and *C* is the number of GMM components. Thus, the EM algorithm is not feasible for real-time operations in large environments. Secondly, the number of GMM components must be given manually at the beginning of the training procedure, which is hard to estimate without prior knowledge. Attempts, such as those involving hierarchical forms, have been widely explored to address these drawbacks [15,16,21,27,28]. Typically, these methods operate “bottom-up”, repeatedly grouping together like clusters of points and using divergence measures to split and merge the data. Goldberger et al. [27] constructed an iterative EM-like algorithm using Kullback–Leibler divergence (KL-Divergence) in order to repeatedly merge candidate clusters. Srivastava et al. [15] initialized the hierarchy by inputting an overestimated amount of Gaussians at the lowest level and iteratively merging similar components measured by KL-divergence. In contrast, Eckart et al. [16] applied a “top-down” hierarchical approach by exploiting the sparsity of the responsibility matrix, achieving high time efficiency through parallel computing.

The similarity of two probability density functions (PDFs) can be measured by adopting various types of divergences or distances. However, not all divergences are equally useful in terms of the GMM. KL-Divergence is the most well-known criterion for mutual information between two probability density functions. While the KL-Divergence was used in [15,27] for merging similar Gaussians, it does not yield an analytic closed-form expression for GMMs. Estimation methods for KL-divergence between GMMs can be found in [29,30,31]. However, the estimation methods are either not accurate or computationally expensive. A closed-form information criterion for the GMM is desired. An analytical solution for the GMM based on Cauchy–Schwarz PDF divergence [32] measurements was derived in [33]. Wang et al. [34] proposed a GMM registration technique by utilizing Jensen–Renyi divergence [35]. Jian et al. [18] and Ma et al. [36] analytically solved the point set registration problem based on L2 distance of the GMM. We choose the Cauchy–Schwarz pdf divergence as the information metric to measure the similarity between GMMs because it resembles KL-divergence.

In this paper, a probabilistic representation of three-dimensional point clouds is developed in the form of a hierarchy of Gaussian Mixture Models. The hierarchy derive from the top-down and coarse-to-fine structure proposed by [16], which eases constraints on point-to-cluster assignments and enables a high time efficiency. To solve the issue that the number of Gaussian components between levels are inconsistent, we introduce information-theoretic measurement as well as distribution shape of the covariances as stop conditions. As a result, the proposed approach can dynamically allocate anisotropic Gaussians to local areas with high-frequency details as well as a simple geometry. The proposed model can recreate original point cloud in multifidelity and arbitrary resolutions, which gives high flexibility in robot applications.

The paper is organized as follows. Section 2 states the theoretical foundation required to develop the proposed approach. Section 3 presents the method of our algorithm. Section 4 gives the details of implementation. A comprehensive evaluation is conducted in Section 5. Finally, the conclusions and Future work are discussed in Section 7.

## 2. Gaussian Mixture Model Representation

### 2.1. Gaussian Mixture Model

In this work, the Gaussian Mixture Model is used as a representation method. The Gaussian Mixture Model is a parametric probability density function represented as sum of Gaussian densities

(1) $$p\left(x|\Theta \right)=\sum_{c=1}^{C}\rho_{c}N\left(x|\mu_{c},\Sigma_{c}\right).$$

N(x|μ,Σ) is the three-dimensional multivariate Gaussian density, whose parameter $\mu \in R^{3},\Sigma \in R^{3*3}$ are the mean vector and the covariance matrix. The scalar $\rho_{c}$ is the weight of the Gaussian component.

Given a three-dimensional point cloud Z of size N, assuming the points within the point cloud are independent and identically distributed (i.i.d) on the surface being modeled, the likelihood of the environment generated by a GMM can be expressed as

(2) $$p\left(Z|\Theta \right)=\prod_{n=1}^{N}p\left(z_{n}|\Theta \right)=\prod_{n=1}^{N}\sum_{c=1}^{C}\rho_{c}N\left(z_{n}|\mu_{c},\Sigma_{c}\right).$$

Since there is no closed form solution for the GMM, the Expectation-Maximization (EM) algorithm [37] is commonly utilized to find an estimation by iteratively maximizing data likelihood. Specifically, by establishing the correspondence between points and Gaussian components of the mixture through a set of binary latent variables, the E-step evaluates the corresponding posterior probability by computing the expectation of latent variables

(3) $$\Gamma \equiv E\left[r_{nc}\right]=\frac{\rho_{c}p\left(z_{n}|\Theta_{c}\right)}{\sum_{i=1}^{C}\rho_{i}p\left(z_{n}|\Theta_{i}\right)},$$

while the M-step maintains the current responsibilities and re-estimates the parameters by maximizing the expected log-likelihood

(4) $$\Theta^{new}=\underset{\Theta }{\mathrm{argmax}}\sum_{n=1}^{N}\sum_{c=1}^{C}\gamma_{nc}\left\{\ln\rho_{c}+\ln N\left(z_{n}|\Theta_{c}\right)\right\}.$$

$E\left[r_{nc}\right]$ is defined as the responsibility matrix Γ, where $\gamma_{nc}$ is the responsibility of point *n* to the $c_{th}$ Gaussian.

The optimization of the M-step can be analytically solved using Equation (5), where $\Gamma_{c}=\sum_{n}\gamma_{nc}$ and $\Theta^{new}=\left\{\rho^{new},\mu^{new},\Sigma^{new}\right\}$ are the updated parameters in each step. By iteratively computing the E-step and M-step, final output can be determined when the average data log-likelihood converge to a threshold.

(5) $$\begin{array}{cc} \mu_{c}^{new} & =\frac{\sum_{n}\gamma_{nc}z_{n}}{\Gamma_{c}},\\ \Sigma_{c}^{new} & =\frac{\sum_{n}\gamma_{nc}z_{n}z_{n}^{T}}{\Gamma_{c}}-\mu_{c}^{new}\mu_{c}^{new^{T}},\\ \rho_{c}^{new} & =\frac{1}{N}\Gamma_{c}. \end{array}$$

### 2.2. Sparsity of Responsibility Matrix

**Figure 1:**
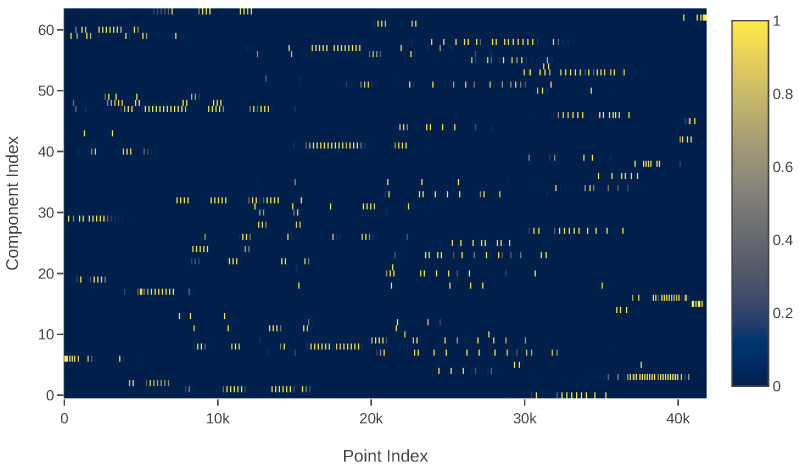
Visualized responsibility matrix. The dark region occupies a significant percentage, meaning the matrix is sufficiently sparse.

The points collected by range sensors are usually distributed along the surfaces of objects. On the basis of the nature of 3D geometry, the posterior over correspondences (responsibilities) are sufficiently sparse when modeling with a large number of Gaussians. Figure 1 visualizes a responsibility matrix of a point cloud whose *x*-axis is points and *y*-axis is the index of the associated mixture component. The corresponding responsibilities are visualized as dark to light colors, where the light colors denote a high contribution to the responsibilities while the dark colors denote the opposite. From the figure, we can see that a high percentage of the matrix is occupied by dark colors, meaning most matrix elements will not contribute meaningfully after the expectation step of the EM algorithm. Consequently, the summation of these zero or nearly zero values in the maximization step will cause a low computational efficiency and finally lead to a poor timing performance.

To address the issue caused by the sparsity of the responsibility matrix, we employ a hierarchical structure that recursively segments the environment into local parts by clustering responsibilities that have a meaningful contribution. The details are discussed in the next section.

## 3. Method

### 3.1. Hierarchy

**Figure 2:**
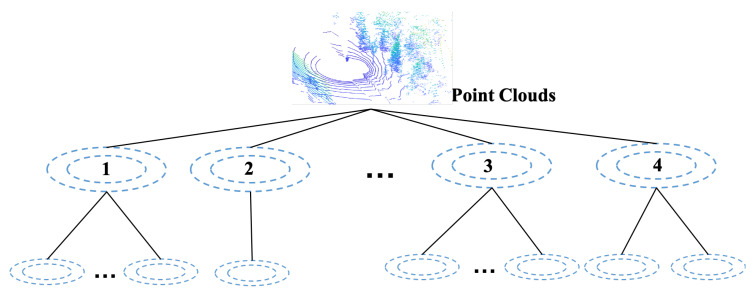
The proposed hierarchical model. The root of the tree is the input point cloud. Each level is an independent Gaussian Mixture Model (GMM). The ellipse denotes weighted Gaussians. Gaussian 1: the parent Gaussian spawns another mixture of size *C* in the normal generation. Gaussian 2: the local point clouds are defined as being well modeled by their distribution shape. Gaussian 3: the children mixtures are determined as being well modeled by the information metric. Gaussian 4: the size of GMM children is cut in half if ill-conditioned covariance is encountered.

**Our approach uses anisotropic weighted Gaussians for three-dimensional environment** representations. By exploiting the sparsity of the responsibility matrix discussed earlier, a hierarchical structure is employed by the small-sized recursive children of the GMMs and the partitioned points by selecting points that have meaningful contributions. In this way, the general EM training with time complexity O(N×C) is divided into a collection of trainings with a decreasing size, leading to a log-speed acceleration. The proposed structure is presented in Figure 2. The root of the hierarchy is input point cloud. Each ellipse represents a weighted Gaussian of the GMM. Each level is an independent GMM that estimates the point cloud. In the hierarchical structure, the point cloud is firstly fitted with a GMM of size *C*. Then, points within the point cloud are associated with the Gaussians. The parent Gaussians spawn more children from another GMM using the associated local point clouds. The default size of the children are set as a fixed positive integer. The structure is a full tree if no condition is set to interfere with the generation. With the increase in level, more Gaussian components are generated, and the point cloud are modeled with higher fidelity. However, the number of Gaussians grows exponentially with the increase in levels, causing a huge gap in the number of fitting Gaussians. The lack of adaptivity easily leads to underfitting and overfitting problems. We propose several conditions that determine if a parent Gaussian should continue to spawn children. For these local details that have been well modeled, the Gaussians are seen as converged and remain static in the following procedures, while the others proceed until all are converged. The converged mixtures are denoted as the children of Gaussian 2 to Gaussian 4 in the figure, and the stop conditions are further discussed in Section 3.3.

In order to maintain a valid global Gaussian Mixture at each level, the correct GMM parameters must be updated after the generation of the children level. Suppose the current training level is *l*, the probability of the GMM in children level l+1 generating the point cloud can be expressed as

(6) $$p\left(z_{i}|G^{\left(l+1\right)}\right)=\sum_{c=1}^{K}\sum_{c^{\prime }=1}^{J_{c}}\rho_{c^{\prime }}^{\left(l+1\right)}\rho_{c}^{\left(l+1)|(l\right)}p\left(z_{i}\left|\Theta_{c}^{\left(l+1)|(l\right)}\right),\right.$$

where $\rho_{c}^{\left(l+1)|(l\right)}$ and $\Theta_{c}^{\left(l+1)|(l\right)}$ are the children parameters of the GMM $G^{\left(l\right)}$, *K* is the number of Gaussians in level *l*, and $J_{c}$ is the number of children refined by the *c*th component in the parent GMM $G^{\left(l\right)}$. The formula shows that in order to update the children model, the weight value must be propagated down from the parent level.

### 3.2. Partition

We use parent responsibility matrices to associate points with child mixtures [16]. Considering the fact that some marginal points may be shared by multiple Gaussians, we introduce a matrix P and two coefficients: $\lambda_{p}^{u}$ and $\lambda_{p}^{l}$. The sufficiently large $\lambda_{p}^{u}$ is used to control the amount of information sharing among children of different parents. While a relatively small $\lambda_{p}^{l}$ deals with the situation that points are not assigned to any child mixture after applying $\lambda_{p}^{u}$. Specifically, for responsibilities greater than $\lambda_{p}^{u}$, the corresponding elements in P are set to 1, while the others remain zeros. For points that do not have significant contributions to any children Gaussian mixtures, the elements fall below $\lambda_{p}^{l}$ are set to zeros, while the others are set to 1. Then, the matrix is normalized by rows. Algorithm 1 describes the procedure in detail. $\Gamma_{n}$ and $P_{n}$ denote the $n_{th}$ row vector of the matrix. Line 12 normalizes P by rows so that the sum of each row is equal to 1. The partition matrix reflects how the local point clouds are assigned to children mixtures. In addition, its values are used as the weight of the points in the successive modeling procedures. When $\lambda_{p}^{u}$ is sufficiently large, only a small amount of points are shared by children mixtures, maintaining the algorithm’s high computational efficiency.
**Algorithm 1** Partition1:**procedure**Partition($\Gamma ,\lambda_{p}^{u},\lambda_{p}^{l}$)2:    **Init:** P←zeros3:    **for all** $\;\Gamma_{\mathrm{nc}}\geq \lambda_{p}^{u}$ **do**4:        $P_{nc}=1$5:    **end for**6:    $idx\leftarrow find(SUM\left(P_{n}\right)==0)$7:    **for all** $idx\in \Gamma_{n}$ **do**8:         **if** $\;\Gamma_{\mathrm{nc}}\geq \lambda_{p}^{l}$ **then**9:           $P_{nc}=1$10:        **end if**11:    **end for**12:    **Return** Normalized(P)13:**end procedure**

### 3.3. Stop Conditions

The amount of Gaussians grows exponentially with the increase in the training level. One simple termination method is set a maximum level. However, the number of Gaussian components between two levels has a gap, and it is hard to find a proper fitting level. In this section, we propose several stop conditions that can adaptively and efficiently terminate the generation when a local point cloud is well fitted.

#### 3.3.1. Distribution Shape

To enable a meaningful Gaussian distribution, the covariance of the Gaussian must be a positive semi-definite matrix. To describe the shapes of the distributions, we specify the eigenvalues and eigenvectors of the covariance matrix as $\sigma =\{\sigma_{1},\sigma_{2},\sigma_{3}\}$ and $v=\{v_{1},v_{2},v_{3}\}$, where we define $\sigma_{1}\geq \sigma_{2}\geq \sigma_{3}$. A covariance matrix can be visualized as a three-dimensional ellipse utilizing v as the orientations and σ as the scale factors on the corresponding axises. Normally, the distribution shapes can be described as planar, linear, and spherical.

We introduce two coefficients, $s_{l}$ and $s_{p}$, where $s_{l}=\sigma_{3}/\sigma_{2}$ and $s_{p}=\sigma_{2}/\sigma_{1}$. Then, a distribution is linear if we have $s_{l}\leq \lambda_{e}$, and planar if it is nonlinear and $s_{p}\leq \lambda_{e}$. If a distribution has no eigenvalue $1/\lambda_{e}$ times larger than another one (nonlinear and nonplanar), then it is spherical. For the reason that the points collected by range sensors are distributed along surfaces, we assume the point cloud in the real world to be locally planar. For the hierarchical structure, when a parent distribution is sufficiently planar, it stops generating new children and is collected by the converged mixture set.

#### 3.3.2. Information Metric

Divergence measurements seek to provide a measure of the distance or the mutual information between two probability distributions. Cauchy–Schwarz Divergence [33] is widely used in probability-based techniques [38,39,40]. The Cauchy–Schwarz PDF divergence measure is defined as

(7) $$CS\left(p,q\right)=-\log\left(\frac{\int q(x)p(x)dx}{\sqrt{\int q{\left(x\right)}^{2}dx\int p{\left(x\right)}^{2}dx}}\right).$$

Unlike KL-divergence, CS-divergence is a symmetric measurement for any two PDFs *p* and *q*, such that 0≤CS(p,q)<∞ and the minimum is obtained when and only when p(x) = q(x). For two Gaussian distributions $N\left(x|\mu_{a},\Sigma_{a}\right)$ and $N\left(x|\mu_{b},\Sigma_{b}\right)$, the integration of the dot product is

(8) $$\int N\left(x|\mu_{a},\Sigma_{a}\right)N\left(x|\mu_{b},\Sigma_{b}\right)dx=N\left(0|\mu_{a}-\mu_{b},\Sigma_{a}+\Sigma_{b}\right),$$

which enables a closed-form solution for the GMM. Given a GMM G of size J and $G^{\prime }$ of size K, the CS-divergence of the GMMs can be obtained using Equation (9), where $\Theta_{j}=\left\{\rho_{j},\mu_{j},\Sigma_{j}\right\}$ and $\Theta_{k}^{\prime }=\left\{\rho_{k}^{\prime },\mu_{k}^{\prime },\Sigma_{k}^{\prime }\right\}$ are the $j_{th}$ and $k_{th}$ Gaussians of G and $G^{\prime }$.

(9) $$\begin{array}{cc} CS(G,G^{\prime })= & -\log\left(\int GG^{\prime }dx\right)+\frac{1}{2}\log\left(\int G^{2}dx\right)+\frac{1}{2}\log\left(\int G^{\prime 2}dx\right),\\ = & -\log\left(\sum_{j=1}^{J}\sum_{k=1}^{K}\rho_{j}\rho_{k}^{\prime }N\left(0|\mu_{j}-\mu_{k}^{\prime },\Sigma_{j}+\Sigma_{k}^{\prime }\right)\right)\\ \; & +\frac{1}{2}\log\left(\sum_{j=1}^{J}\rho_{j}^{2}\frac{1}{{\left(4\pi \right)}^{\frac{D}{2}}{\left|\Sigma_{j}\right|}^{\frac{1}{2}}}+2\sum_{j=1}^{J}\sum_{j^{\prime }<j}\rho_{j}\rho_{j^{\prime }}N\left(0|\mu_{j}-\mu_{j^{\prime }},\Sigma_{j}+\Sigma_{j^{\prime }}\right)\right),\\ \; & +\frac{1}{2}\log\left(\sum_{k=1}^{K}\rho_{k}^{\prime 2}\frac{1}{{\left(4\pi \right)}^{\frac{D}{2}}{\left|\Sigma_{k}^{\prime }\right|}^{\frac{1}{2}}}+2\sum_{k=1}^{K}\sum_{k^{\prime }<k}\rho_{k}^{\prime }\rho_{k^{\prime }}^{\prime }N\left(0|\mu_{k}^{\prime }-\mu_{k^{\prime }}^{\prime 2},\Sigma_{k}^{\prime }+\Sigma_{k^{\prime }}^{\prime }\right)\right). \end{array}$$

Since the mixtures in the hierarchy are probability distributions, we apply CS-divergence as a metric to measure the mutual information between GMMs. In the proposed approach, we focus on the similarity between parent Gaussians and their children. In the case that local estimation made by a parent Gaussian is not accurate enough, the children mixtures further refine it with higher fidelity, causing a considerable divergence between parent and children GMMs. Oppositely, if a local environment is well fitted by a Gaussian mixture, not much information is gained by continuous generation, leading to a small value of divergence. This is the case on the basis that a threshold parameter $\lambda_{cs}$ is introduced to determine if a local part of a point cloud is well fitted. Specifically, the CS-divergences between the parent and its children are computed. For those that fall below $\lambda_{cs}$, the children stop generating and are collected by the converged mixture set.

To deal with ill-conditioned covariances, regularization methods may be applied within the training process, which may have an impact on the metric. To avoid this issue, in the case in which the covariance matrix is ill-conditioned, the EM fitting makes an attempt with half the number of training Gaussian components. In the case that only two Gaussians remain, The ill-conditioned covariances can be regularized.

## 4. Implementation

### 4.1. M-Step Vectorization

When implementing the EM algorithm, other than for loops, the M-step can be easily rewritten using matrix multiplication. Algorithm 2 shows the procedure. Vector w is the weights of points derived from matrix P. We denote $\Gamma_{c}$ as being the $c_{th}$ column of Γ. The superscript *T* is the transpose operation of the matrix. The *FLAT* operation reshapes a column-major matrix to a row vector. Thereafter, the *SUM* operation sums up all of the vector elements. By rewriting the algorithm, it is more feasible to achieve a high time efficiency when implementing it in different programming languages and deploying it through different computation platforms.
**Algorithm 2** M Step Rewrite1:**procedure**M_Step(Z,Γ,w)2:    **for all** $z_{n}$ **do**3:        $X_{n}\leftarrow FLAT\left({z_{n}}^{T}z_{n}\right)$4:    **end for**5:    **for all** c∈C **do**6:        $\rho_{c}\leftarrow \Gamma_{c}w/SUM\left(w\right)$7:        $\mu_{c}\leftarrow \left(\Gamma_{c}^{T}Z_{d}\right)/\rho_{c}$8:        $\Sigma_{c}\leftarrow FLAT\left({\Gamma_{c}}^{T}X_{d}/\rho_{c}\right)-FLAT\left(\mu_{c}^{T}\mu_{c}\right)$9:    **end for**10:   **Return** ρ,μ,Σ11:**end procedure**

### 4.2. Implementation

Algorithm 3 presents the pseudocode of our implementation. At each level, the global GMM $G^{\left(l\right)}$ is composed of two sets, $G_{converged}$ and $G_{training}$, where the Gaussians in $G_{training}$ are responsible for refining local parts into higher fidelity, while $G_{converged}$ collects the converged mixtures determined by the stop conditions. If a mixture is marked as converged, it stops generating children, making it a static component of the global GMM. After training of each level, the Gaussians in $G_{training}$ are substituted with the refined children mixtures, and both sets propagate to the next level. This process continues until all the Gaussians in level *l* are collected by the $G_{converged}$, where we have $G^{\left(l\right)}=G_{converged}$, then the procedure is terminated and the hierarchy M is returned as the final output.

At the beginning of the procedure, only point cloud data and the number of children are necessarily given as inputs. $\Theta_{init}$ is used as initial input for every EM train. The mean vectors are set to be the points sampled evenly within the point cloud. The covariances are set as fixed diagonal matrices. The mixing weights are initially equal. The $G_{converged}$ is initially empty and $G_{training}$ is composed of a GMM of size *C*. Line 8 to 15 shows the refining process of partitioning point cloud using the EM algorithm. The local point clouds and the point weight vector w are extracted from the partition matrix. As illustrated earlier, w is used to prevent multiple counts. The E_Step computes the responsibility matrix using Equation (3) while the M_Step optimizes the parameters by maxing the log-likelihood function. In our approach, the M_Step is calculated using our rewritten Algorithm 2.

To model environments with an adaptive number of weighted Gaussians, the proposed converge conditions are utilized. The distribution shape is determined in Line 5. We assume that point clouds are locally planar. If a Gaussian satisfies $s_{p}\leq \lambda_{e}$, it will be seen as sufficiently planar and collected by $G_{converged}$. Furthermore, the CS-divergence is utilized to find mutual information between parents and their children (line 16 to 19). The divergences that fall below $\lambda_{cs}$ show that parents and their children have a high level of similarity. Then, the children mixtures are collected by the converged GMM set.

After training of each level, the Partition procedure is implemented based on the responsibility matrices to give a proper association to the local point clouds, as illustrated in Algorithm 1. The global GMM of each level is the collection of $G_{training}$ and $G_{converged}$.
**Algorithm 3** Hierarchical Training1:**procedure**HierarchicalModel(Z,C)2:    **Init:** $\Theta_{init},G_{converged}\leftarrow empty,G_{training}\;$3:    **while** $!\;empty(G_{training})$ **do**4:        **for all** $\;m\in G_{training}$ **do**5:           **if** $s_{p}\leq \lambda_{e}$ **then**6:               $G_{converged}\leftarrow G_{converged}\;\cup \;\Theta_{m}$7:           **else**8:               $c^{\prime }\leftarrow \;$ C, $\{Z_{m},w\}\leftarrow P$9:               **while** status!=succeed **do**10:                   **while** !converged**do**                ▹ EM algorithm11:                       $\left\{\Gamma_{m},status\right\}\leftarrow E\_Step\left(Z_{m},\Theta_{\mathrm{init}},c^{\prime },w\right)$     ▹ Equation (3)12:                       $\Theta_{children}\leftarrow M\_Step\left(\Gamma_{m}\right)$              ▹ Algorithm 213:                   **end while**14:                   $c^{\prime }\leftarrow c^{\prime }/2$15:               **end while**16:               **if** $\;CS\left(\Theta_{m},\Theta_{children}\right)\leq \lambda_{cs}$ **then**17:                   $G_{converged}\leftarrow G_{converged}\;\cup \;\Theta_{children}$18:               **else**19:                   $G_{training}\leftarrow \;\Theta_{children}$20:               **end if**21:           **end if**22:        **end for**23:        $P\leftarrow Partition(\Gamma ,\lambda_{p}^{u},\lambda_{p}^{l})$                 ▹ Algorithm 124:        $G^{\left(l\right)}\leftarrow G_{training}\cup G_{converged}$25:    **end while**26:    **Return** $M=\{G^{\left(1\right)},G^{\left(2\right)},...G^{\left(l\right)}\}$27:**end procedure**

## 5. Evaluation

To evaluate the performance of our approach, we introduce several public datasets. The **Corner** and **Office** are from the TUM (https://vision.in.tum.de/data/datasets/rgbd-dataset) dataset [41], whose range data are collected by a Microsoft Kinect RGB-D camera. The **Corner** sets consist of grounds and walls in a clear area. The **Office** is an office scene which might be commonly found in a disorganized indoor environment. The **Street** dataset is a subset of the NCLT (http://robots.engin.umich.edu/nclt/) dataset [42] collected via a 32-channel Lidar in an outdoor environment. The **Campus** dataset is a subset of the KITTI (http://www.cvlibs.net/datasets/kitti/raw_data.php) dataset [43] collected via a 64-channel Lidar in an outdoor environment. These datasets are composed of structured walls, pedestrians, as well as scattered foliage. Detailed information is listed in Table 1.

To give a comprehensive evaluation, we compare three state-of-the-art approaches for comparison. Hereafter, the Expectation-Maximization algorithm stated in [44] is referred to as EM. The bottom-up approach proposed in [45] is referred to as HGMM. The top-down approach proposed in [16] is denoted as GMM-Tree. Those three approaches are all based on the EM algorithm. NDT is a widely used state-of-the-art model proposed in [5] that also can be seen as a mixture of Gaussians. For the HGMM, we set the initial GMM size to 300. For the GMM-Tree, we set the number of children to eight and the maximum components as $8^{4}$. For our approach, we set C=8, $\lambda_{p}^{u}=0.35$.

### 5.1. RGB-D Dataset

**Figure 3:**
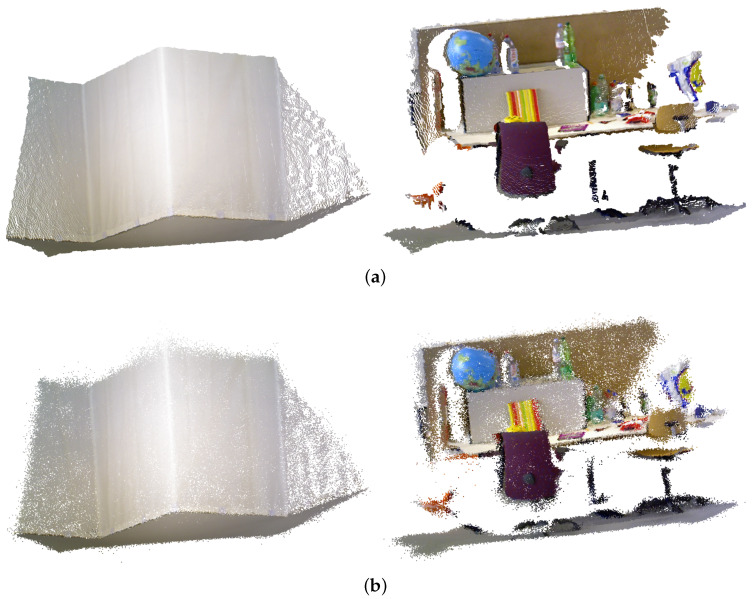
The raw point clouds of the RGB-D datasets and the reconstructed point clouds by our approach. (**a**) Raw point cloud; (**b**) Reconstruction using our approach.

The Gaussian Mixture Model is continuous parametric distribution. Thus our approach can recreate environments by resampling an arbitrary amount of points [44]. Figure 3 illustrates an example of raw RGB-D data selected from the datasets as well as their reconstructed point clouds of different approaches. The point cloud size is consistent with the size of the input point clouds.

The figures demonstrate that our approach provides a fairly good approximation. For example, in the **Office** linear parts such as chair legs are reconstructed in a high resolution, the curve surfaces of the globe are also recreated, showing that our approach has the ability to dynamically allocate Gaussians to the details throughout the environment. Detailed analysis of fidelity is discussed in the following section.

#### 5.1.1. Fidelity

The fidelity provides a measure between the original environment and the GMM representation. We utilize a Peak Signal to Noise Ratio (PSNR) metric to evaluate the representation fidelity. Specifically, given a reference point cloud of size N, we generate an equivalent N amount of points from its corresponding GMM representation. For every point in the original point cloud, we find the nearest neighbor in the reconstructed cloud. Then, the mean square error (MSE) of point clouds is obtained. The PSNR is computed by

(10) $$PSNR=10log_{10}\left(\frac{p^{2}}{MSE}\right),$$

where *p* is the diagonal distance of the bounding box of the original point cloud [46].

**Figure 4:**
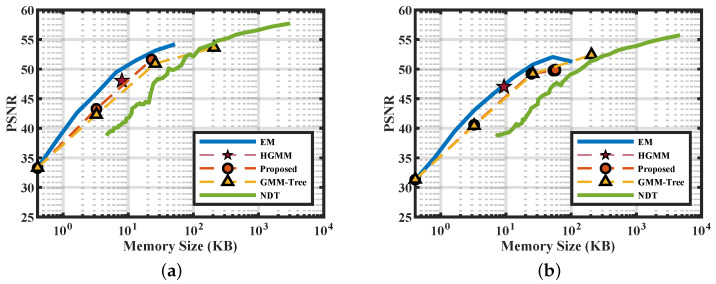
Peak Signal to Noise Ratio (PSNR) of the scenes selected from the RGB-D datasets. (**a**) Corner; (**b**) Office.

Figure 4 gives the PSNR with respect to the memory size of the scenes shown in Figure 3a. The *x*-axis is plot in log scale. We set the number of Gaussian components of the EM distribute within the log space. From the figure we can clearly see the trade-off between memory size and fidelity. Apart from adaptive approaches like the HGMM and our approaches, the others can seek better PSNR by trading memory. The curves of the EM in linear space is convex. We define the term “knee-zone” as the converging area beyond where the fidelity will not vary significantly. Seeking fidelity beyond the “knee-zone” is not cost-effective for most of the models. For example, in Figure 4a, the fidelity gain of the GMM-Tree in level four is only two extra compared to level three, but level four costs eight times more memory. Thereafter, the data located within the knee-zone can be seen as a balance between fidelity, memory efficiency, and fitting time. For all RGB-D scenes, the final results of our approach (the endpoint of the red curves) fall within the knee-zone of the curves, validating the efficiency of our approach. As a result of the need for voxelization, the PSNR of the NDT is lower than ours at similar memory sizes.

#### 5.1.2. Efficiency

**Figure 5:**
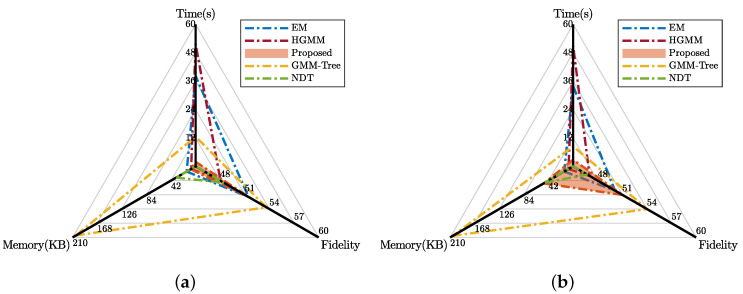
The mean value of fitting time, fidelity, and memory footprint of RGB-D datasets using different approaches. (**a**) Corner; (**b**) Office.

The efficiency of our approach is investigated in this section. For the RGB-D datasets, we computed the mean values of the fitting time, fidelity, and memory footprint of different approaches. The grid size of the NDT is set to 0.2 m. The fitting Gaussians of the EM is set to 300. The results are plotted in Figure 5.

From the figure, we can see that our approach has the best efficiency compared to the other approaches. Our approach uses an efficient method to control generation by dynamically allocating Gaussian mixtures based on the information and local geometry. If we set the EM as a baseline, our approach greatly saves the fitting time while providing a comparable fidelity. The HGMM achieves adaptivity by computing the KL-divergence between Gaussian mixtures and merging similar Gaussians. The extra computation of KL-divergence worsens the time performance. And the HGMM’s PSNR is slightly lower than that of the EM because of the merging process. Its advantage is in memory footprint. The GMM-Tree takes advantage of decoupling the sparse responsibility matrix, therefore achieving a high time efficiency compared to EM and HGMM. Even though the GMM-Tree has the best performance in terms of fidelity, however, as a result of lacking adaptivity, it has the worst memory efficiency, which the memory occupancy is several times greater than ours. And the consumption of computation also leads to a worse time performance. Due to no need to iteratively approximate solutions, the NDT is the fastest among all approaches. In spite of that, our approach has better performance in terms of the other two aspects. In Figure 5a our approach outperforms NDT in both memory and fidelity. In Figure 5b our approach and the NDT has similar memory but our approach achieves better fidelity.

### 5.2. Lidar Dataset

**Figure 6:**
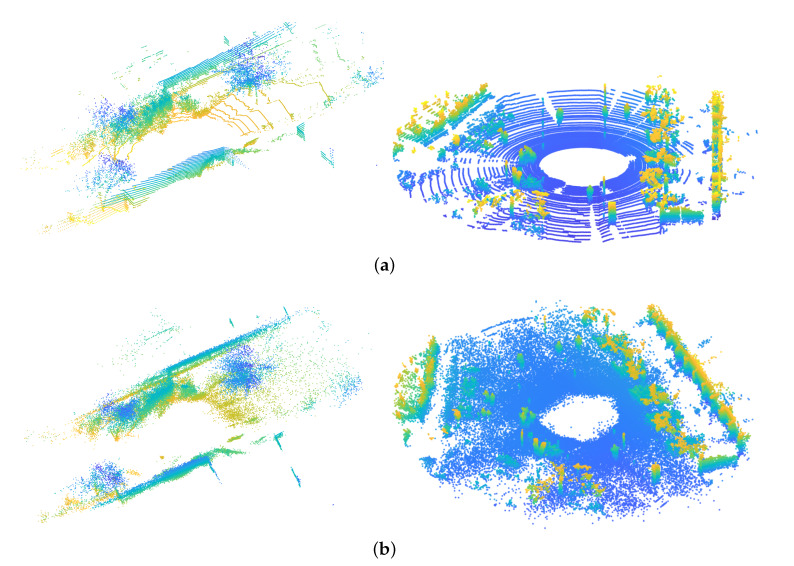
The raw point clouds of the Lidar datasets and the reconstructed point clouds by our approach. (**a**) Raw point cloud; (**b**) Reconstruction using our approach.

Figure 6 illustrates the examples of raw point clouds as well as their reconstructed point clouds of the Lidar datasets. However, as a result of the characteristics of the Velodyne LiDAR sensor, the data points between the two scan channels are inconsistent. For example, instead of forming a dense point cloud as is the case with RGB-D cameras, the points along surfaces distribute as dot lines. When employing GMM-based approaches, the gap between two laser scans can end up being filled up (see the grounds and walls of the reconstructed point clouds). Even though these models normally reflect the ground truth of the geometrical world, to avoid extreme cases that may lead to a false conception, we control the absolute size of the Gaussians in our method so that the representation can achieve a certain resolution.

#### 5.2.1. Fidelity

As a result of the characteristics of the Velodyne LiDAR sensor, the Point-to-Point PSNR does not reflect the real performance of GMM-based approaches. Consequently, we redefine the MSE as the mean square of Point-to-Surface error to evaluate the Lidar datasets. Specifically, for every point in an original point cloud, its local plane is estimated using six neighboring points. Then, for the resampled point cloud, the MSE with respect to the local plane is computed by

(11) $$MSE=\frac{v\cdot (z_{n}^{r}-z_{n})}{\left|v\right|},$$

where $z_{n}$ is the point of the original point clouds, v is the normal vector of $z_{n}$, and $z_{n}^{r}$ is a point of the resampled point cloud. Then, the PSNR is finally computed using Equation (10).

**Figure 7:**
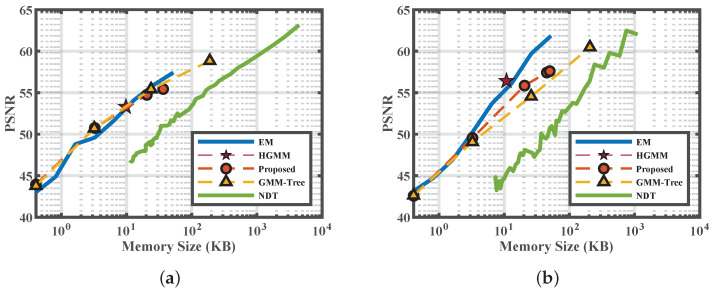
Peak Signal to Noise Ratio (PSNR) of the scenes selected from the Lidar datasets. (**a**) Street; (**b**) Campus.

Figure 7 gives the PSNR with respect to the memory size of the scenes shown in Figure 6a. The situation is slightly different from the RGB-D datasets. In Figure 7b, instead of apparently converging with the increase of fitting Gaussians, the EM curves show a linear increase in log space. We conclude this is caused by the sparsity of the large portion of foliage, where laser hits are randomly scattered. The EM and NDT show their strength in modeling point cloud into higher fidelity. Nonetheless, the final mixture of our approach gives a comparable fidelity to the EM with 300 components.

#### 5.2.2. Efficiency

**Figure 8:**
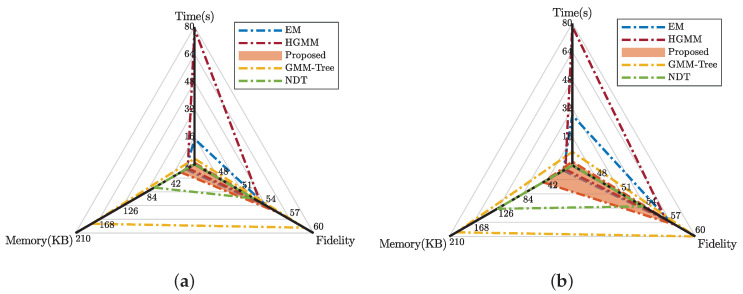
The mean value of fitting time, fidelity, and memory footprint of Lidar datasets using different approaches. (**a**) Street; (**b**) Campus.

The performance of different approaches in terms of fitting time, fidelity, and memory footprint are illustrated in Figure 8. We set the NDT’s grid size to 1.5m. Other parameters are consistent with the RGB-D datasets.

The results are consistent with the RGB-D datasets. In terms of timing, our approach gives an average of 15 times acceleration compared to the EM benefit from the partition, and three times acceleration compared to the GMM-Tree from adaptivity. The GMM-Tree achieves a better PSNR for a sufficient number of fitting Gaussians, while our approach finds a balance between time cost, memory efficiency, and modeling fidelity. With respect to the NDT, our approach is superior in fidelity and memory.

## 6. Discussion

Section 5 gives comparisons of our approach against other state-of-the-art approaches. In this section, we will discuss the real performance of our approach in the applications of robotics. The proposed approach can be leveraged to facilitate robots in several ways. Intuitively, as a parametric distribution, the GMM can resample an arbitrary amount of points. Then a grid map can be generated using these points. The created grid map can be further utilized in navigation. To assess the grid map constructed by the resampled points, we take the map created by raw point cloud as ground truth, then query the status of the two maps with a set of uniformly distributed points. The results of the True Negative Rate (TNR) and False Positive Rate (FPR) are shown in Table 2. The grid maps created by our approach show high resemblance compared to the original point clouds’, where for most of the cases, the overall positive rates are over 98%. The True Negative Rates, which are hazardous when enabling navigation, only account for a very small percentage of the error rates.

Another application of our approach is robot localization through registration. Given a point cloud Z and a GMM representation G, the rigid transformation *T* can be obtained by maximizing the data probability using Equation (12), where the transform *T* can be iteratively solved using the Expectation-Maximization method [21].

(12) $$\hat{T}=\underset{T}{\mathrm{argmax}}p\left(T\left(Z\right)|G\right)$$

**Figure 9:**
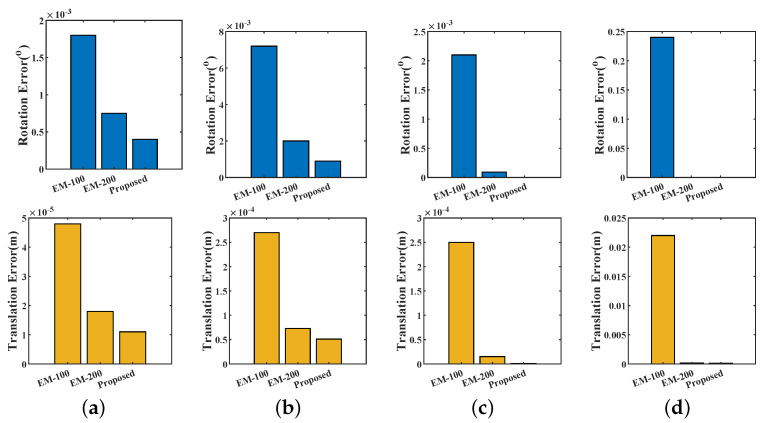
The rotation errors and translation errors of the GMM registrations.EM-100 and EM-200 are the EM-trained GMM using 100 and 200 components, respectively. (**a**) Corner; (**b**) Office; (**c**) Street; (**d**) Campus.

For the scenes shown in Figure 3a and Figure 6a, we first fit them with GMM representations. Then the raw point clouds are rotated and translated along all their degree of freedom. We conduct registrations using the transformed point clouds with respect to their GMM representation. The errors are shown in Figure 9. The figures show that the GMMs short of components lead to higher errors. And the influence of the number of components on Lidar datasets is more than RGB-D datasets. However, we found that if a great amount of GMM components were used, the cost function would oscillate when iteratively maximizing. Thus the registration will take many more steps (therefore more time) to converge, and it is easier to fall into local minima. Thereafter, a proper number of fitting components is desired. Our approach presents its strength in the adaptive fitting.

## 7. Conclusions

In this paper, a 3D point cloud representation based on the Gaussian mixture model is proposed. By replacing a large EM problem with multiple smaller ones, the proposed hierarchical structure significantly accelerates training speed. The mutual information and shape of the probabilistic distributions are introduced to determine if a local environment is well represented, leading to full adaptivity in allocating weighted Gaussians. The evaluations demonstrate that our approach can effectively model the point cloud and be applied in robotic with high efficiency.

## Figures and Tables

**Table 1:** Detailed information of the datasets.

	Corner	Office	Street	Campus
**Sensor**	RGB-D Camera	RGB-D Camera	LiDAR	LiDAR
**Model**	Microsoft Kinect	Microsoft Kinect	Velodyne HDL-32E	Velodyne HDL-64E
**Source**	TUM	TUM	NCLT	KITTI
**Scene**	Indoor	Indoor	Outdoor	Outdoor
**Structure**	Simple	Complex	Medium	Complex
**Valid Size**	∼2.91 MB	∼2.54 MB	∼0.61 MB	∼1.22 MB

**Table 2:** The True Negative Rate (TNR) and False Positive Rate (FPR) of different datasets with respect to different grid sizes.

Grid Size	0.3 m		0.2 m		0.1 m	
	**TNR**	**FPR**	**TNR**	**FPR**	**TNR**	**FPR**
**Corner**	0.44%	1.33%	0.27%	1.37%	0.24%	0.99%
**Office**	0.29%	0.86%	0.15%	1.44%	0.24%	1.19%
**Street**	0.70%	1.34%	0.46%	0.78%	0.13%	0.19%
**Campus**	1.20%	3.79%	1.01%	2.50%	0.52%	0.84%
